# Research on Absolute Positioning Sensor Based on Eddy Current Reflection for High-Speed Maglev Train

**DOI:** 10.3390/s20185167

**Published:** 2020-09-10

**Authors:** Xiaobo Hong, Jun Wu, Yunzhou Zhang, Yongxiang He

**Affiliations:** College of Intelligent Science, National University of Defense Technology, Changsha 410073, China; hongxiaobo@163.com (X.H.); zyz_ss1210@163.com (Y.Z.); heyongxiang1995@163.com (Y.H.)

**Keywords:** maglev train, absolute positioning sensor, eddy current reflection, position marker plate, code-reading reliability

## Abstract

A novel absolute positioning sensor for high-speed maglev train based on eddy current effect is studied in this paper. The sensor is designed with photoelectric switch and four groups of unilateral coplanar code-reading detection coil combination. The photoelectric switch realizes the positioning of the marker plate, and the four groups of detection coils read the mileage code of the mileage sign plate to obtain the absolute mileage information of the vehicle, which effectively reduces the quality and volume of the sensor, and reduces the impact of ice and snow. At the same time, the code-reading reliability and speed adaptability index are proposed. The code-reading reliability of the sensor is analyzed and tested under the fluctuation of levitation guidance, and the positioning error under the speed range of 0–600 km/h is calculated and analyzed. The results show that the novel sensor has the advantages of simple and compact structure. It still satisfies the system’s requirements for absolute vehicle mileage information under the conditions of vehicle operating attitude fluctuations and changes in the full operating speed range.

## 1. Introduction

High-speed maglev train is driven by linear synchronous motor and operates without contact with track by electromagnetic force suspension [1,2]. In order to realize accurate positioning and speed closed-loop control during driving, the speed measurement and positioning system is necessary to be installed on the maglev train [3,4,5]. The existing speed and position measurement for maglev train include velocity measurement and absolute positioning based on radar [6,7], velocity measurement and relative positioning based on cross induction loop [8,9,10], absolute positioning based on query transponder, absolute positioning based on pulse width coding, absolute positioning based on electromagnetic induction, velocity measurement, and absolute positioning based on inductive wireless communication [11,12,13,14].

**Figure 1:**
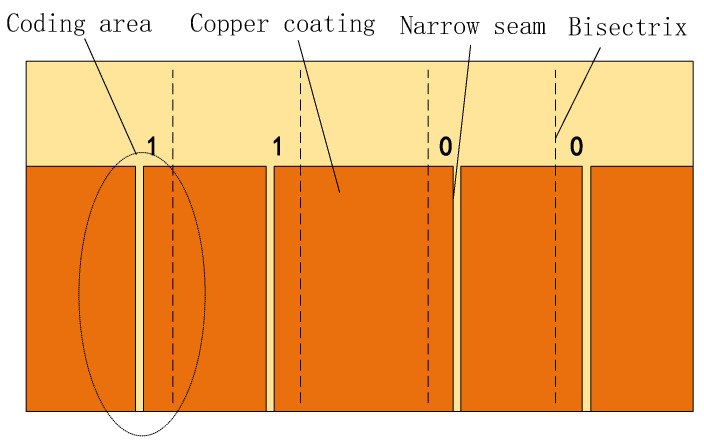
The structure of the position marker plate.

At present, the absolute positioning method based on electromagnetic induction is adopted for high-speed maglev train. Its working principle is to scan the passive position marker plate (hereinafter referred to as the marker plate) along the track through the onboard absolute positioning sensor, so as to obtain the position information [15,16,17]. The position code on the marker plate is realized by special treatment on the copper coating of the marker plate. Each marker plate has a four-bit binary code, and the position of the narrow seam relative to the five-bisector of the marker plate indicates that the bit code is 1 or 0. As shown in Figure 1, the code is 1100.

**Figure 2:**
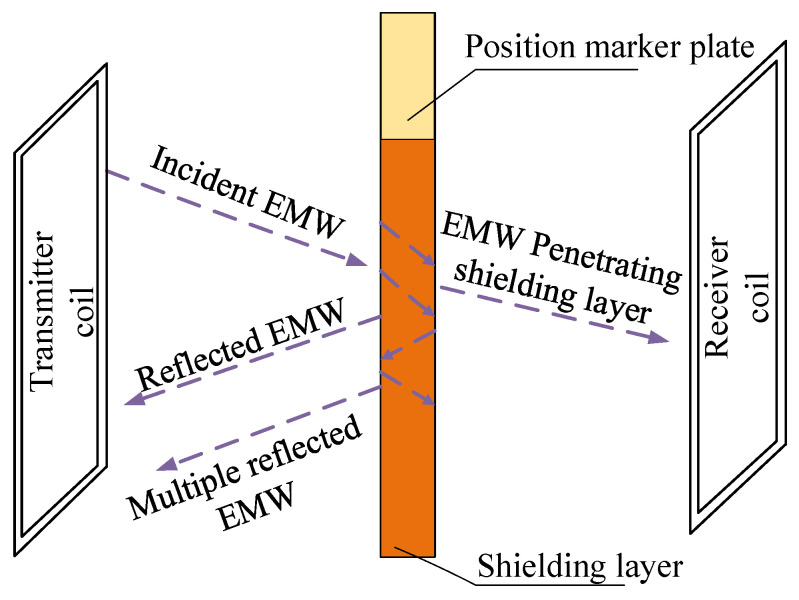
Schematic diagram of electromagnetic wave penetrating the position marker plate.

The copper layer of the marker plate can shield the magnetic field. Copper has a high conductivity, and the high-frequency magnetic field can be offset by the eddy current reverse magnetic field generated by the copper coating [18,19]. As shown in Figure 2, the electromagnetic wave (EMW) emitted by transmitter coil passes through the copper coating layer, the copper coating layer will absorb part of the electromagnetic wave, and the remaining electromagnetic wave will penetrate the shielding layer and be received by receiver coil. As the absolute positioning sensor currently used in high-speed maglev train, INK (the sensor was first developed by Germany, so the German name INK is still used in the field of maglev, which is the abbreviation of INKREFA_Messeinheit in German) uses the transmission detection principle, and adopts symmetrical transmitter coil and receiver coil to transmit and detect electromagnetic wave respectively. In the narrow seam of the marker plate, the incomplete copper coating leads to more electromagnetic wave transmission. Accordingly, the sensor will receive more electromagnetic waves at the seam, so as to identify the seam and read the position code of the marker plate [20,21].

In this paper, a new type of absolute positioning sensor (Hereinafter referred to as NINK, add ***N*** before INK to represent the new type) based on eddy current reflection is studied. NINK uses photoelectric switch to position, and four groups of coplanar coils arranged in the same plane are used to detect the eddy current reverse magnetic field of the shielding layer of the marker plate. Therefore, NINK can read the four-bit code of the marker plate at the same time. Compared with INK, NINK changes the coil layout, positioning, and code-reading mode, and reduces the number of coils, so that the sensor has the advantages of simplified structure, small size, and light weight. In addition, it reduces the risk of collision between the sensor and the marker plate. In addition, whether NINK can adapt to the running environment of high-speed maglev trains is the focus of this paper.

The remaining parts of this paper are arranged as follows: Section 2 introduces the structure and working principle of NINK. In Section 3, the code-reading reliability index and speed adaptability index are proposed to evaluate the influence of vibration on code-reading result and the influence of speed on code-reading reliability. In Section 4, the working performance of NINK is tested and analyzed, which verifies the working performance of NINK under the conditions of vehicle suspension and guiding fluctuation. In Section 5, the platform is used to test the output of NINK at different detection distances and attitude angles, and the comparison is made with INK. At last, Section 6 concludes this paper.

## 2. Working Principle and Realization of NINK

### 2.1. INK

**Figure 3:**
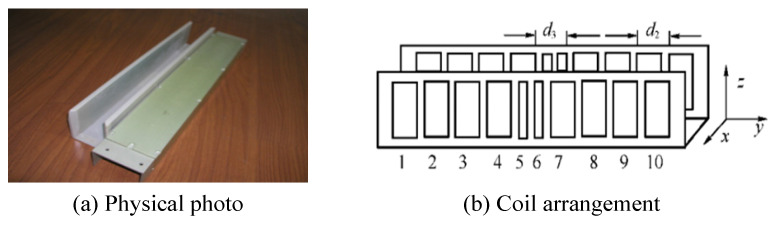
INK and its coil arrangement.

INK adopts the transmission detection principle, and its structure is U-shaped, as shown in Figure 3a. In Figure 3b, the transmitter coil and receiver coil of the sensor are arranged symmetrically on both sides [22,23], with a total of 10 groups. Eight groups of wide coils are used as positioning coils (1–4 and 7–10 in Figure 3), and two groups of narrow coils (5 and 6 in Figure 3) are code-reading coils, which are used to read the address code of the marker plate. Positioning error refers to that the detection coil deviates from the ideal reading position. Constrained by the dynamic response of the positioning coil, the positioning error is large in high-speed detection, which is easy to lead to code-reading error. Therefore, the code-reading value is generally determined by comparing the terminal voltage of two code-reading coils.

**Figure 4:**
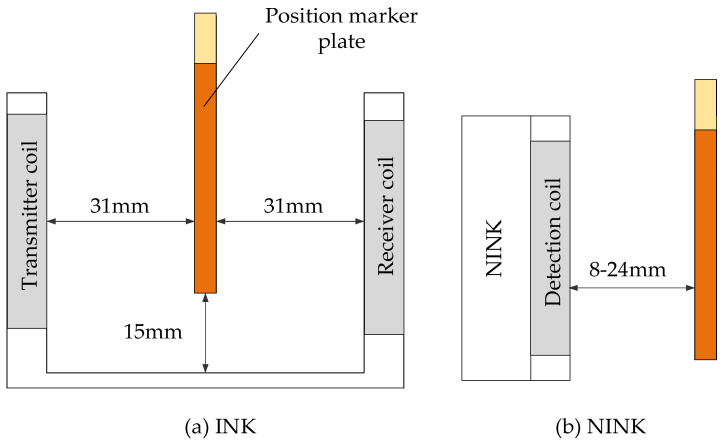
The relative position between the marker plate and the two sensors.

U-shaped structure, coil positioning mode and sequential code-reading mode lead to a large number of coils, correspondingly large volume, and mass. When INK works, the marker plate passes through the U-shaped slot, as shown in Figure 4a. The width of u-shaped slot is 66 mm, and the distance from the bottom to the lower surface of the marker plate is 15 mm. Within this size range, the marker plate can successfully pass through the U-shaped slot to realize code-reading without being affected by vehicle vibration and attitude change. However, in extreme bad weather conditions, such as freezing rain, if the surface of the marker plate has thick ice, especially when the bottom surface is covered with ice edges, the marker plate may collide with sensor and cause damage. Obviously, changing the U-shaped structure can reduce the risk of collision. In addition, if the positioning or reading mode of the sensor can be changed and the number of coils can be reduced, the structure of the sensor can be simplified, and the volume and mass can be correspondingly reduced.

### 2.2. NINK Positioning Sensor Design and Relization

**Figure 5:**
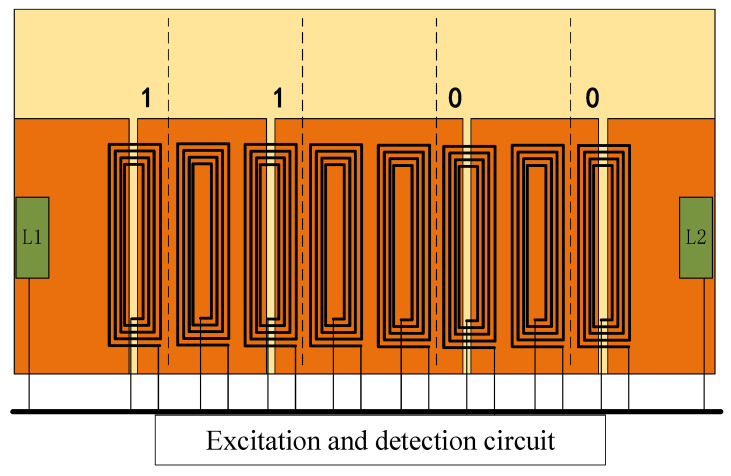
The coil layout of NINK.

In view of the shortcomings of INK, this paper proposes an absolute positioning sensor NINK based on eddy current reflection. NINK utilizes the principle of eddy current reflection, and the detection coil can both receive and emit electromagnetic waves. So only one surface of NINK is the detection surface, as shown in Figure 4b. Different from the U-shaped structure, NINK has no vertical motion restriction relative to the marker plate, which can avoid the collision between the sensor and the marker plate in extreme environments. As shown in Figure 5, the eddy current generated in the copper coating reduces the equivalent inductance of the coil, while the narrow seam can block the eddy current. By measuring the change of the coil terminal voltage, whether the detection object is a narrow seam can be identified. In order to determine the position of the seam relative to the bisector, a comparison detection coil is needed. By comparing the voltage of the two coils, the relative position of the seam can be determined, and the code of the positioning marker plate can be read. In order to read the four-bit code of the marker plate, eight detection coils are arranged in the same plane. Each two coils are a code bit in a group of corresponding codes, and the inductance value and geometric size of the two coils in the same group should be consistent as far as possible.

**Figure 6:**
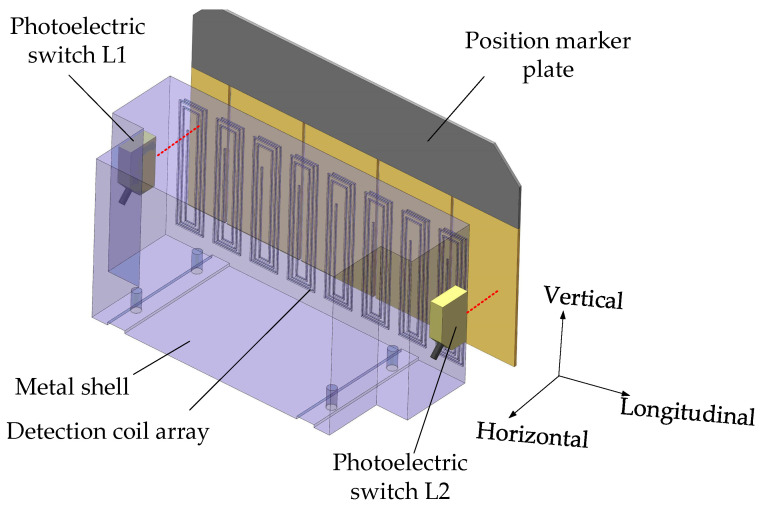
The working state of NINK.

Photoelectric switch is used to locate, compared with coil positioning, optoelectronic switch has higher dynamic response characteristics, which can ensure that NINK has a lower response delay; thus, reducing the positioning deviation. The working state of NINK is shown in Figure 6, photoelectric switches L1 and L2 are used to determine whether the absolute positioning sensor reaches the intended reading position. L1 and L2 are arranged on both sides of the detection coil, and the distance between them is less than the length of the marker plate. When the sensor enters the detection range from the forward direction, L1 and L2 will be successively placed ON state. If L1 and L2 are on at the same time, it means that the sensor is facing the marker plate, indicating that NINK is located in the reading position, and then output the current reading value.

Since the coils of NINK have the functions of transmitting and receiving electromagnetic waves, and all coils are arranged in the same plane, only one side of the NINK is the detection surface, so NINK can avoid contacting with the sign plate vertically, which greatly reduces the risk of collision. NINK uses photoelectric switch to replace the positioning coil, which reduce positioning deviation caused by response delay. In addition, NINK can read four-digit code simultaneously with four sets of detection coils. Compared with INK, the number of coils is reduced from 20 to 8, so its structure is simpler, and its size and mass are smaller. At present, the length of NINK prototype is about 300 mm and the mass is about 3 kg, which is significantly lower than INK. With those advantages of compact structure and small volume and weight, NINK can be installed in pairs on the train to achieve the purpose of redundancy configuration, which provides a new solution for absolute mileage detection of high-speed maglev train.

**Figure 7:**
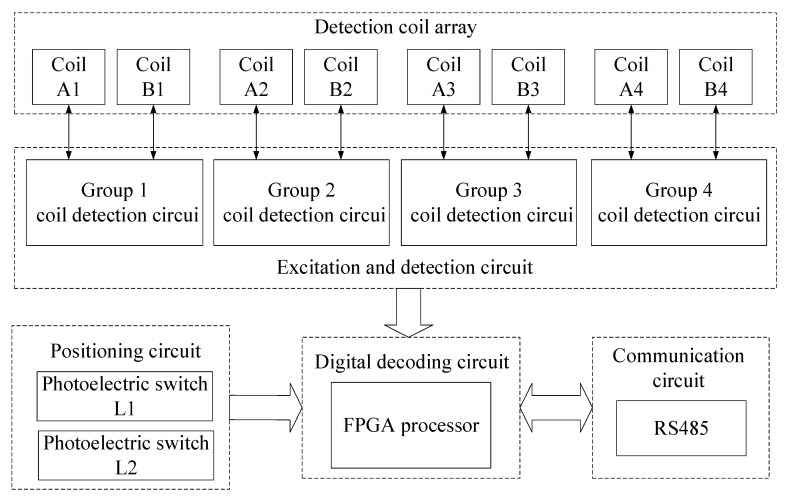
The circuit structure of NINK.

**Figure 8:**
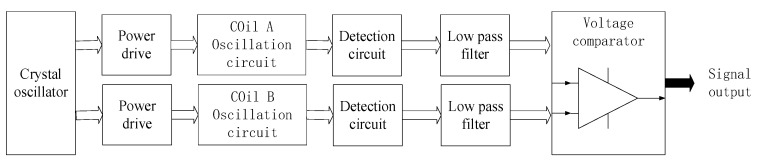
The workflow of the excitation and detection circuits of the coils.

The circuit structure of NINK is shown in Figure 7. The detection coil array of NINK is divided into 4 groups to detect the coding of the corresponding position of the marker plate, respectively. Each group of coils has its own excitation and detection circuits to excite the coils to generate high-frequency vibration and to detect the peak-to-peak voltage of each coil. Figure 8 shows the working flow of the excitation and detection circuits of a group of coils, the oscillating circuits of the two coils share a crystal oscillator to ensure that the two coils work synchronously. The detection and filter links can obtain the peaking value of the coil terminal voltage after removing clutter interference, and feed the two coil voltage values into the voltage comparator, judge the position of the narrow seam of the marker plate by comparing the voltage, and output the corresponding voltage signal. Four groups of coil detection circuits send the voltage comparison results to the FPGA processor for decoding, so as to determine the marker plate code and analyze the current position. Finally, the sensor sends the decoded information to the upper computer through RS485 communication circuit, and the position detection is completed after confirmation by the upper computer. In addition, the filter capacitor is connected to the input terminal of the power module of NINK, and the input/output cables are shielded to suppress EMI interference. The aluminum case also makes the sensor have good electromagnetic compatibility.

## 3. Performance Evaluation Index of NINK

The correct address code output by NINK is very important for train operation and control. When the maglev train is running, the levitation and guidance fluctuation will cause the detection coil of NINK to deviate from the ideal position when reading code, which may cause wrong code-reading. Because the output of NINK has only two states: right and wrong, it can be considered that the output of NINK has no concept of error, so the general performance evaluation index in sensor field cannot be directly applied to NINK. In this section according to the working principle of NINK, two indexes are proposed to evaluate the performance of the sensor, which can assist its design and test. First of all, this section puts forward the code-reading reliability index to evaluate the impact of vehicle body vibration on the reliability of code-reading results. Then, for the positioning error caused by vehicle speed change, the speed adaptability index is proposed to quantify the impact of vehicle speed on code-reading reliability.

### 3.1. Code-Reading Reliability Index

By comparing the peak voltage of the two detection coils, NINK can determine the relative position of the narrow seam; thus, reading the address code of the marker plate. However, due to the levitation and guidance fluctuation of maglev train during operation, the position of NINK relative to the marker plate is dislocated up and down, and the detection distance changes; thus, changing the equivalent inductance of the detection coil. These influencing factors make the detection result of the sensor uncertain, so the code-reading reliability index $c_{rel}$ is proposed to make a quantitative evaluation of the reliability degree of the detection result.

Let the central line position of coil *A* be $x_{A}$, the Central Line position of coil *B* be $x_{B}$, the center distance between the two coils be $x_{A-B}$, and the voltage difference between coil *A* and coil *B* be ΔU:

(1) $$\Delta U=\left|U(x_{A})-U(x_{B})\right|$$

**Figure 9:**
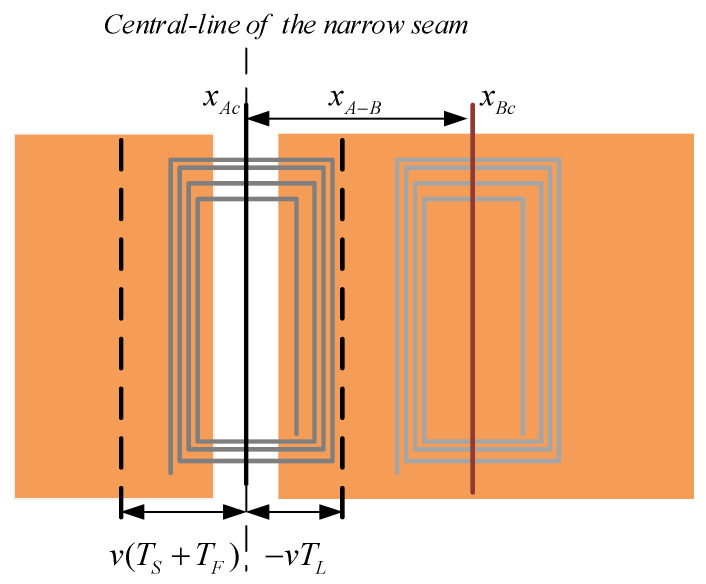
Schematic diagram of coil position.

The larger the voltage difference ΔU is, the more obvious the feature of the narrow seam is, indicating the sensor has higher code-reading credibility. It is known from experience that the peak value of coil terminal voltage is the highest when the coil is facing the narrow seam, and the lowest when the coil is facing the complete copper coating. As shown in Figure 9, the position of coil *A* facing the narrow seam is $x_{Ac}$, and the position of coil *B* is $x_{Bc}$, then the maximum value of the voltage difference is

(2) $$\Delta U_{\max}=\left|U(x_{Ac})-U(x_{Bc})\right|$$

In practice, the voltage difference ΔU may be too small due to the influence of external interference magnetic field, detection circuit error, detection distance or position offset exceeding the limit, etc. If ΔU fluctuates near zero, the output of the voltage comparator changes frequently, causing the processor to mistakenly decode. Therefore, NINK uses a voltage comparator with hysteresis characteristics, whose output signal only jumps when ΔU reaches the voltage threshold of the comparator. Using this kind of voltage comparator can compare the voltage state of coil *A* and *B* more accurately; thus, improving the reliability of the output results. The reliability index of code-reading is defined as $c_{rel}$:

(3) $$c_{rel}=\frac{\Delta U_{\max}-V_{HYST}}{V_{HYST}}$$

where $V_{HYST}$ is the hysteresis voltage of the comparator, and the hysteresis voltage in this paper is 80 mV.

According to Equation (3), $c_{rel}$ greater than 0 indicates that the test result is reliable. The larger *C* is, the more reliable the reading code is. The larger the $c_{rel}$, the higher the reliability of code-reading.

### 3.2. Speed Adaptability Index

The impact of train running speed (detection speed) on code-reading reliability must be considered since NINK is installed on maglev trains. Due to the response time of NINK’s photoelectric switch, oscillation circuit, detection circuit and filter circuit, the delay in code-reading process is inevitable. Because the sensor works in motion state, the system delay can be expressed in spatial scale, which is called geometric delay. The higher detection speed is, the greater the geometric delay will be. Record the geometric delay as $x_{delay}$:

(4) $$x_{delay}(v)=v(T_{S}+T_{F}-T_{L})$$

where $T_{S}$, $T_{F}$, $T_{L}$ are respectively the response time of oscillation circuit, filter circuit, and photoelectric switch.

Geometric delay is manifested as positioning error of NINK, which is the deviation between the actual reading position and the standard reading position. The standard reading position is the position when the center line of the detection coil coincides with the center line of the narrow seam,$T_{L}$ causes the actual read position to be ahead of the standard position, while $T_{F}$ and $T_{S}$ mean that the actual read position lags behind the standard position, Figure 9 shows the intuitive expression of the geometric delay. The geometric delay causes the detection coil to deviate from the standard read position, which in turn causes the equivalent inductance and terminal voltage of the coil to change. In order to quantify the impact of speed on code-reading reliability, a speed adaptability index, $c_{hsa}$, is proposed:

(5) $$c_{hsa}=\frac{\left|U(x_{Ac}\pm x_{delay})-U(x_{Bc}\pm x_{delay})\right|-V_{HYST}}{V_{HYST}}$$

In order to meet the maximum detection speed of 600 km/h, speed adaptability index $c_{hsa600}$ is specifically defined:

(6) $$c_{hsa600}=\frac{\left|U(x_{Ac}\pm x_{dv600})-U(x_{Bc}\pm x_{dv600})\right|-V_{HYST}}{V_{HYST}}$$

where, $x_{dv600}$ represents the geometric delay of the system when the detection speed is 600 km/h. Considering the dynamic response characteristics of photoelectric switch, oscillation circuit, detection circuit and filter circuit, the total delay of NINK in this paper is about 40 μs, by substituting the total delay into Equation (4), the geometric delay under 600 km/h can be obtained as follows:

(7) $$x_{dv600}=x_{delay}(167\;m/s)=6.7\;\mathrm{mm}$$

When NINK is in the maximum geometric delay, if the ratio of coil voltage difference ΔU to hysteresis voltage $V_{HYST}$ is large, it indicates that NINK’s code-reading reliability is still high, and it is considered that the sensor has good high-speed adaptability within the detection speed range of 600 km/h.

## 4. Performance Analysis of NINK

Since the working performance of NINK is affected by train suspension, guidance fluctuation and running speed, the code-reading reliability and speed adaptability indexes proposed in Section 3 are used to evaluate the working performance of the sensor. In order to analyze the working performance of NINK in the case of vehicle suspension and guidance fluctuation, the equivalent inductance and terminal voltage change of the detection coil are simulated and tested, and the performance is quantified based on the code-reading reliability and speed adaptability index. Finally, the code-reading reliability of NINK under different detection distance and attitude changes is verified by using the test platform.

**Figure 10:**
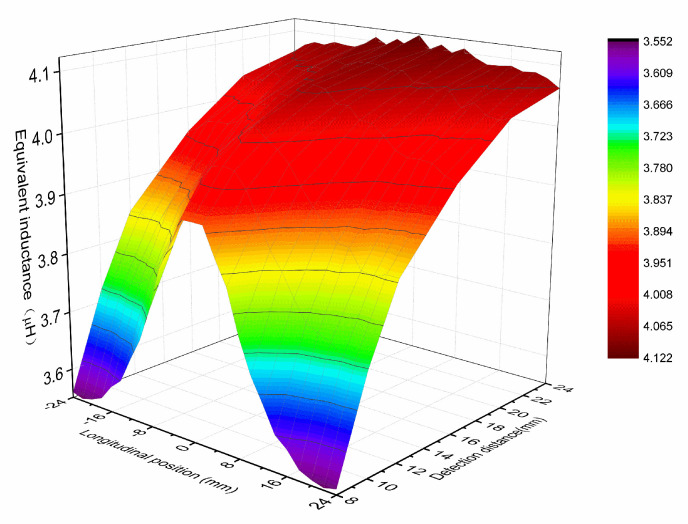
The equivalent inductance of the detection coil at different longitudinal positions and detection distances.

In the narrow seam position of the sign board, the detection coil works in an asymmetric working state because the detection surface is an incomplete copper coating surface. It is complex to solve the inductance and voltage parameters of the coil by using the analytical method. Therefore, the finite element simulation and actual measurement are used for simple analysis. This paper first simulated the equivalent inductance of the detection coil under different longitudinal positions and detection distances, as shown in Figure 10. Zero millimeters represents the position where the detection coil is aligned with the narrow seam of the marker plate (the position of coil A in Figure 9, $x_{Ac}$), where the equivalent inductance of the coil is the largest. The equivalent inductance is larger when the detection distance is larger, and the equivalent inductance is up to 4.12 μH when the detection distance is 24 mm. In addition, if the detection distance is increased, the curve of equivalent inductance changes with longitudinal position becomes flat.

**Figure 11:**
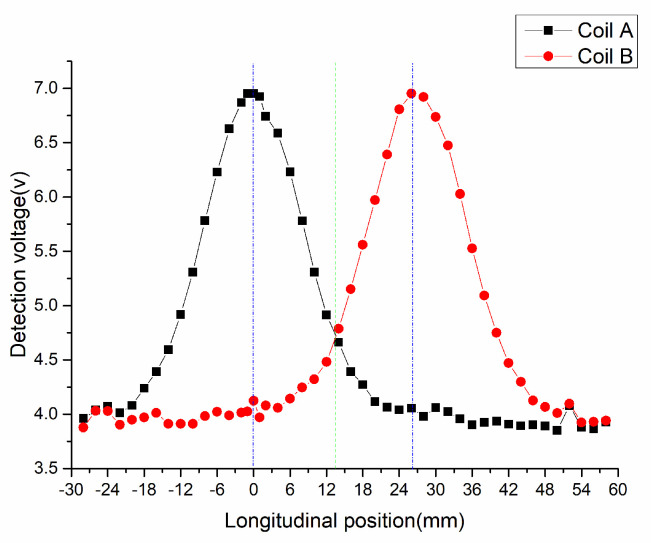
Voltage of detection coil varies with longitudinal position (detection distance is 10 mm).

In order to show the working performance of NINK more intuitively, the actual measurement data are used to explain the change of detection coil voltage with positioning error, detection distance and suspension fluctuation. Figure 11 shows the variation of the terminal voltage of coil A and B along with the longitudinal position when the detection distance is 10 mm. The two voltage variation curves are arched, which are consistent with the equivalent inductance variation characteristics. In this figure, positions 0 mm and 26 mm (blue dotted line) correspond to positions $x_{Ac}$ and $x_{Bc}$, respectively, where coil A and coil B have the maximum terminal voltage. However, the terminal voltage of the two coils at 13 mm (green dotted line) is nearly equal, and NINK cannot accurately read the code at this position due to the inability to compare the size of the two voltage.

**Figure 12:**
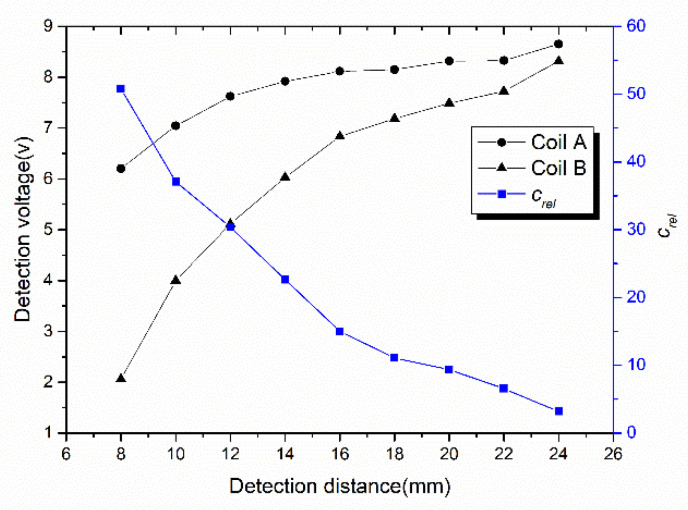
Change of the terminal voltage and the code-reading reliability at different detection distances.

The voltage variation of coils *A* and *B* at different detection distances is shown in Figure 12. Since coil A is facing the narrow seam and coil B is facing the complete copper coating, the voltage of coil A is always greater than that of coil B. Large voltage difference is helpful to identify the narrow seam of the marker plate. When the detection distance is 8 mm, the voltage difference is the largest, so the characteristics of the narrow seam can be fully confirmed. The greater the detection distance is, the closer the voltage of the two coils will be, resulting in the decrease of the code-reading reliability index. When the detection distance is 8 mm, the code-reading reliability is as high as 52, while when the detection distance is 24 mm, the code-reading reliability is less than 5. Obviously, reducing the detection distance can increase the voltage difference and thus increase the code-reading reliability. However, the detection distance should be greater than 8 mm in order to avoid collision between the sensor and the marker plate.

**Figure 13:**
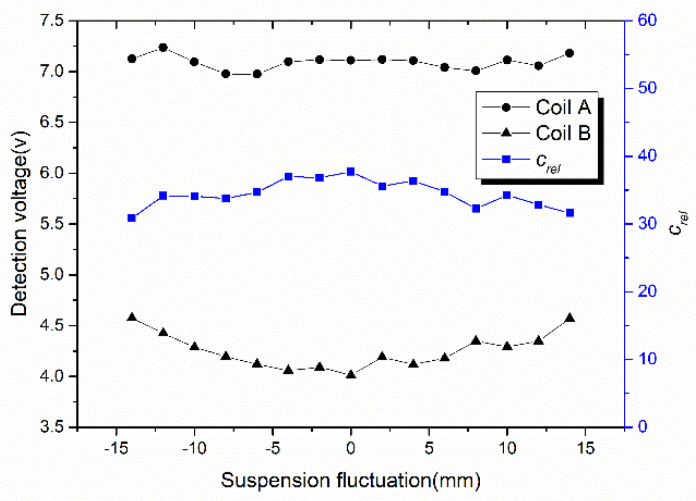
Change of the terminal voltage and the code-reading reliability caused by suspension fluctuation (at detection distance of 10 mm).

Affected by the suspension fluctuation of maglev train, the terminal voltage change of NINK’s detection coils is shown in Figure 13. The voltage of the two coils varies little with the suspension fluctuation, but the voltage difference is always large. The code-reading reliability is all higher than 30, which indicates that the sensor is not significantly affected by the suspension fluctuation of the train.

**Figure 14:**
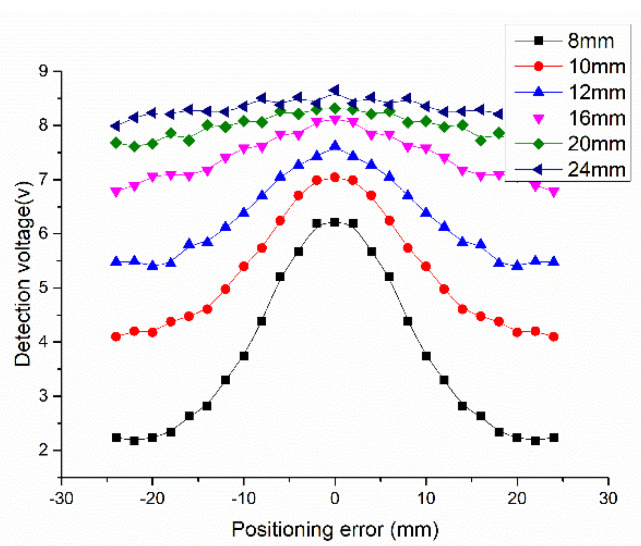
Variation of the terminal voltage caused by positioning error at different detection distances.

**Figure 15:**
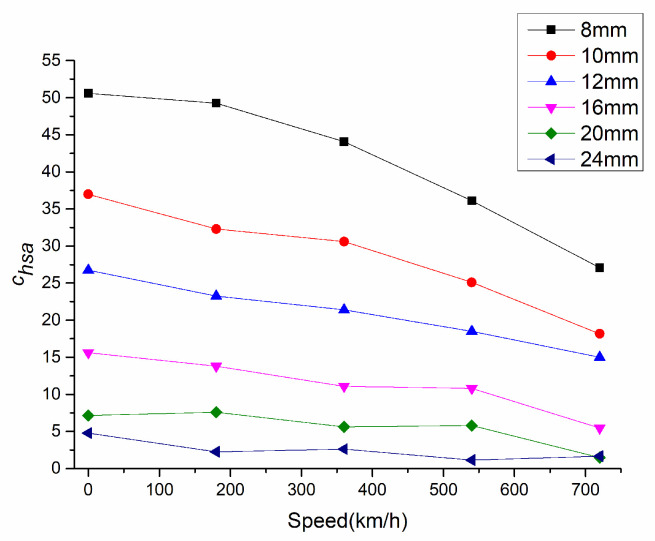
Change of the speed adaptability with the speed at different detection distances.

**Figure 16:**
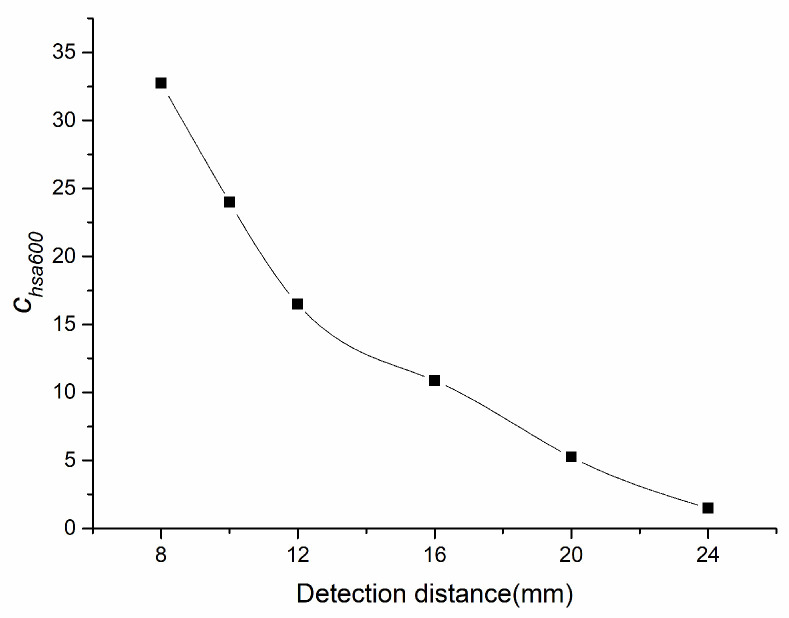
Change of the speed adaptability of 600 km/h at different detection distances.

Due to the geometric delay of NINK with the change of speed, different positioning errors are caused. Figure 14 shows the relationship between the positioning error and the terminal voltage of the coil A. When the positioning error increases, the terminal voltage decreases, and correspondingly, the speed adaptability of NINK also decreases at high speed, as shown in Figure 15. Detection distance has great influence on speed adaptability. At the detection distance of 24 mm, the speed adaptability is lower than five. In this case, although the code can be read correctly, the reliability is not high. Figure 16 shows the changes of speed adaptability of 600 km/h at different detection distances. The speed adaptability at detection distance of 24 mm is close to one. Therefore, it is considered that the detection distance should not exceed 20 mm.

## 5. Testing of NINK

**Figure 17:**
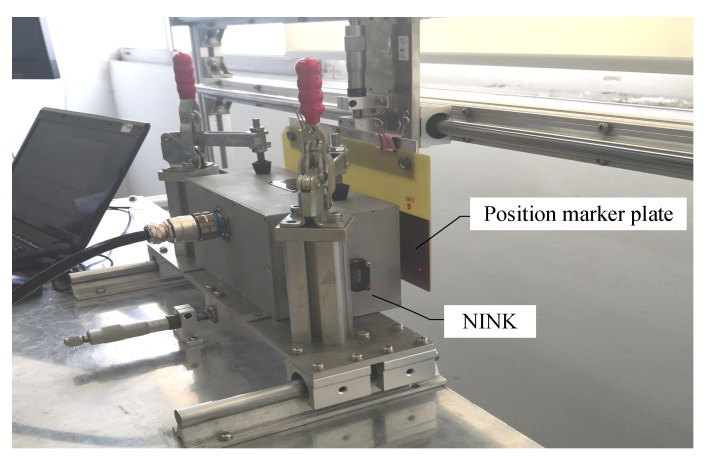
Sensor test platform.

**Figure 18:**
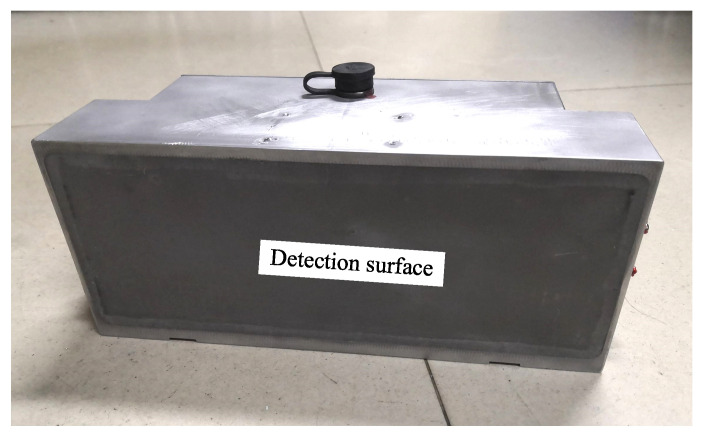
Prototype of NINK.

It can be seen from the above analysis that the guidance fluctuation has a greater impact on the code-reading reliability than the suspension fluctuation. In order to test NINK’s performance during train guided vibration, NINK is fixed on the test platform for simulation test, as shown in Figure 17 (The prototype of NINK is shown in Figure 18). Change the distance between the marker plate and NINK’s detection surface, test and read the output signal. By comparing with the real code of the marker plate, the result of the sensor is judged to be accurate. Test results for INK are also listed in the table to compare with NINK.

The test results of the two sensors at different detection distance are shown in Table 1, which shows that NINK can read the code accurately within the detection distance of 20 mm. Since the guidance fluctuation range of the train is ±2 mm, the detection distance of NINK is 10–14 mm correspondingly. Therefore, NINK can meet the requirements of accurate code-reading under normal train operation conditions. Through comparison with INK, it is concluded that the performance of NINK is approximately close to INK.

Whether it can adapt to the change of train attitude is also an important index to evaluate the working performance of NINK. Due to the change of train attitude, the angle between NINK’s detection surface and the marker plate changes, and the detection distance between each coil and the marker plate also changes; thus, affecting the code-reading reliability. In this paper, the test results of NINK under different attitude angles are given directly in Table 2. Compared with the roll Angle and pitch Angle, the sensor is more affected by the change of yaw Angle. According to the results in the table, NINK can correctly read the code within a yaw Angle of 1° and detection distance of 20 mm. When the yaw Angle is 2° degrees and the detection distance is less than 16 mm, NINK can also read the code correctly. Compared with INK, NINK is more sensitive to the change of train attitude angle. However, as the attitude angle of the train is usually less than 2°, NINK can still work well when the train is in normal operation.

## 6. Conclusions

This paper proposes an absolute positioning sensor based on eddy current reflection. The sensor uses photoelectric switch to realize the location of the marker plate, and four sets of coils are used to detect the eddy current reverse magnetic field of the marker plate shielding layer, which can read the four-bit address code of the marker plate. It has the advantages of simple structure, small body, and light weight. The complexity of the structure is obviously better than the existing absolute positioning sensor. In order to evaluate the influence of train suspension and guidance fluctuation and the working performance of NINK at different speeds, the code-reading reliability and speed adaptability indexes are proposed, and the performance of the sensor is tested and analyzed by using these two indexes. The conclusions are as follows:

(1) The guidance fluctuation of the train has a great impact on code-reading reliability. When the detection distance is 8 mm, the reliability is as high as 51, while the reliability is less than 5 when the detection distance is 24 mm.
(2) The suspension fluctuation of the train has a small impact on the code-reading reliability, and the reliability is higher than 30 when the detection distance is 10 mm.
(3) With the increase of speed, the positioning error of NINK increases, reducing the speed adaptability of the sensor. Especially when the detection distance is large, the speed adaptability is lower.
(4) Through the platform test, it is verified that NINK can accurately read the code when the train’s attitude changes, and when the guidance fluctuates. Comparing the test results with INK, it shows that the performance of NINK is approximately equal to INK under normal train operation conditions, which indicates that NINK can adapt to the operation environment of high-speed maglev train.

## Figures and Tables

**Table 1:** Results of the test at different detection distance.

Position Marker Plate Binarie	NAPS		INK	
	Detection Distance	Conclusion	Lateral Offset	Conclusion
1111B	12 mm	OK	0 mm	OK
0101B		OK		OK
1001B		OK		OK
0001B		OK		OK
1111B	16 mm	OK	+4 mm	OK
0101B		OK		OK
1001B		OK		OK
0001B		OK		OK
1111B	20 mm	OK	+8 mm	OK
0101B		OK		OK
1001B		OK		OK
0001B		OK		OK

**Table 2:** Test results under different attitude angles.

	Attitude	Angle	Detection Distance			
			8 mm	12 mm	16 mm	20 mm
NAPS	Yaw	1°	OK	OK	OK	OK
		2°	OK	OK	OK	ERROR
		3°	OK	ERROR	ERROR	ERROR
	Pitch	1°	OK	OK	OK	OK
		2°	OK	OK	OK	OK
		3°	OK	OK	OK	OK
	Roll	1°	OK	OK	OK	OK
		2°	OK	OK	OK	ERROR
		3°	OK	OK	ERROR	ERROR
INK	Yaw	15°	\	\	\	OK
